# Ovarian clear cell carcinoma meets metabolism; HNF-1β confers survival benefits through the Warburg effect and ROS reduction

**DOI:** 10.18632/oncotarget.5228

**Published:** 2015-08-31

**Authors:** Masaki Mandai, Yasuaki Amano, Ken Yamaguchi, Noriomi Matsumura, Tsukasa Baba, Ikuo Konishi

**Affiliations:** ^1^ Department of Gynecology and Obstetrics, Kyoto University Graduate School of Medicine, Kyoto, Japan; ^2^ Department of Obstetrics and Gynecology, Kinki University Faculty of Medicine, Osaka-Sayama, Japan

**Keywords:** ovarian clear cell carcinoma (OCCC), HNF1β, cancer-specific metabolism, oxidative stress, Warburg effect

## Abstract

Ovarian clear cell carcinoma (OCCC) constitutes one of the subtypes of ovarian cancers, but it has unique clinical, histological and biological characteristics, one of which is chemo-resistance. It is also known to develop from endometriotic cyst, a benign ovarian tumor, at relatively high frequency. Recently, it is becoming well known that most of OCCCs express HNF1β, a transcription factor, which is closely associated with the development of liver, pancreas and kidney, as well as occurrence of familial forms of type 2 diabetes. Expression of HNF1β is now regarded as a hallmark of this tumor. Nevertheless, exact biological function of this gene in OCCC has not been clarified. We have shown in previous studies that microenvironment in endometriotic cysts contains severe oxidative stress and OCCC develops under such stressful environment as stress-resistant tumor, which may lead to chemo-resistance. We also showed that increased expression of HNF1β facilitates glucose uptake and glycolysis, which is known as Warburg effect. In the previous issue of this journal, by using comprehensive metabolome analysis, we report that HNF1β actually reduces and protects themselves from internal oxidative stress by dramatically changing cellular metabolism. In this article, we review the relevance and significance of cancer-specific metabolism and how they are associated with biological characteristics of OCCC via expression of HNF1β, along with future clinical implications of targeting cancer-specific metabolism.

## INTRODUCTION

Ovarian clear cell carcinoma (OCCC) has recently been considered as a distinct entity among epithelial ovarian cancers. While a majority of the epithelial cancers are relatively sensitive to chemotherapy, OCCCs are mostly chemo-resistant [1-4], and a novel therapeutic approach is urgently desired. Other notable features that distinguish this tumor from other subtypes includes frequent occurrence in endometriotic cysts, relatively slow growth, and frequent complication of thromboembolism [4-7]. In particular, the vast majority of cancers arising in endometriotic cysts are either clear cell or endometrioid subtype, suggesting that a unique carcinogenic process is involved [8-10].

Nevertheless, the underlying mechanisms to explain the uniqueness of OCCC are not fully understood, partly because specific genetic events associated with OCCC had not been clarified. However, recently, genetic events including the ARID1A mutation [11-13], activation of the PIK3A mutations [14, 15] and hepatocyte nuclear factor 1β (HNF1β) overexpression [16-18] have been linked to OCCC. We have been continuously investigating the causal relationship between the microenvironment in the endometriotic cyst and the occurrence of OCCC from various viewpoints [8, 10, 19, 20]. Recently, we have shown that HNF1β overexpression significantly influences the metabolic activity of OCCC, which is distinct from that of other ovarian cancers [21]. It has also been suggested that altered metabolism caused by HNF1β overexpression in OCCC is responsible for some of the biological features of OCCC [22]. In this review, we provide an overview of cancer-specific metabolism, discuss how they play important roles in OCCC, and possible future direction of metabolism-targeted cancer therapy.

## MALIGNANT TUMOR EXHIBITS UNIQUE METABOLISM, CALLED WARBURG EFFECT

It has long been recognized that cancer cells have unique, somehow curious metabolic features. In 1924, Otto Warburg reported that cancer cells, unlike most normal cells, prefer to metabolize glucose by glycolysis rather than oxidative phosphorylation, even in the presence of a sufficient oxygen supply that enables cells to perform the latter [22, 23]. Energy production by glycolysis is far less efficient than by oxidative phosphorylation; only 2 ATPs per glucose versus 36 ATPs, respectively, are required [23]. This paradoxical phenomenon is called the “the Warburg effect”. The Warburg effect, which is manifested by increased glucose consumption, decreased oxidative phosphorylation and accompanying lactate production, has been confirmed and is generally accepted as it has been demonstrated in various tumors. It is presently considered to be a metabolic hallmark of cancer [24-26]. This nature of cancers is clinically used as FDG-PET (F-fluoro-2-deoxy-D glucose positron emission tomography) in various cancers including ovarian cancer. It has been reported that tumor metabolic activity is associated with prognosis of the patient with ovarian cancers in general [27] and OCCC [28], although the latter was reported to have lower FDG uptake compared with serous ovarian cancers [29]. However, the causal mechanism and implication for cancer biology still remains unclear.

Recently, several mechanisms have been proposed for the altered metabolism in cancer cells. First, specific genetic events were linked to the change in their metabolic status. In glioblastoma cells, inhibition of H-Ras resulted in diminished glycolysis and cell death [30]. The oncogenic molecule Akt also stimulates aerobic glycolysis in cancer cells [31]. C-Myc potently enhances the glycolytic pathway, increasing target gene expression from glucose transporters through pyruvate kinase [32, 33]. Mutation of the p53 tumor suppressor causes increased glucose consumption by enhancing the pentose phosphate pathway [34]. Therefore, transformation caused by oncogenes often accompanies the alteration of glucose metabolism. Second, the tumor microenvironment sometimes provides pressure for metabolic change, as shown in next section.

## MICROENVIRONMENT-ASSOCIATED GENE INDUCTION IN OCCC INCLUDES HNF1β

**Figure 1:**
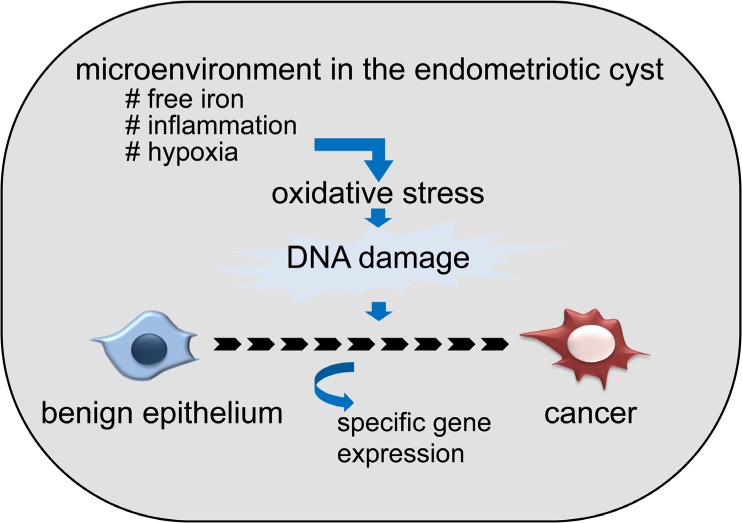
Possible contribution of unique microenvironment in endometriotic cyst to the frequent development of ovarian cancer Microenvironment, especially high concentration of free iron, which derived from old blood accumulated in the cyst, causes oxidative stress (ROS), and causes DNA damage. Accumulation of DNA damage over the years eventually leads to the cancer development.

It is clinically well known that OCCC arises predominantly from ovarian endometriotic cyst, which is characterized by repeated bleeding into the cyst cavity during the menstrual cycle [10]. We have shown that the content of an endometriotic cyst, consisting of old blood, which contains a markedly high concentration of free iron compared with other benign ovarian cysts [19]. Free iron is a source of reactive oxygen species (ROS) and is associated with cancer development through the induction of persistent oxidative stress in several organs, such as the liver and lung [35-37]. In fact, we have found that the level of oxidative stress and the extent of DNA damage were significantly elevated in endometriotic cysts [19]. These data indicate that 1) extensive oxidative stress in the endometriotic cyst may contributes to high incidence of cancer occurrence (Figure 1), and endometriotic epithelial cells which are exposed to extensive oxidative stress must cope with it to survive.

To further investigate the role of microenvironment in OCCC, we employed comprehensive gene-expression analyses. Microarray analysis identified a gene signature that distinguishes clear cell carcinoma from other types of ovarian cancer, which we designated as the ovarian clear cell carcinoma signature (OCCC signature) [20]. Importantly, the OCCC signature was induced when ovarian epithelial cells were exposed to the contents of endometriotic cysts, implying that it is originally driven by the unique microenvironment. A categorical analysis demonstrated that the OCCC signature is mainly comprised of genes belonging to three categories, stress response, sugar metabolism and coagulation, suggesting that these three entities define the biological identity of OCCC.

Among the genes included in the OCCC signature, we focused on HNF1β for the following reasons. First, HNF1β is overexpressed in the majority of OCCC. [16-18]. Due to its sensitivity and specificity, HNF1β overexpression is regarded as a hallmark of OCCC among epithelial ovarian cancers. Second, we have shown that the expression of HNF1β is epigenetically regulated [20, 38, 39]. In comparison of methylation profiles between OCCC and non-clear cell ovarian cancers, a majority of the OCCC-specific hypomethylated genes were shown to have HNF1-binding sites. Third, a pathway analysis of the OCCC signature suggested that HNF1β is a central mediator of the OCCC-specific signaling network [20]. Finally, a motif analysis revealed that HNF1 binding motifs are significantly enriched in genes that comprise the OCCC signature [18]. Collectively, we assumed that HNF1β plays a central role in the manifestation of the unique biological phenotype of OCCC.

## HNF1β IS A KEY FACTOR BOTH IN ORGAN DEVELOPMENT AND CANCER OCCURRENCE

HNF1β is a multifunctional transcription factor that shares strong homology with HNF1α and regulates wide variety of gene expressions [40]. It is especially associated with the developmental process of important organs. HNF1 genes were originally identified as genes preferentially expressed in hepatocytes, and the lack of HNF1β lead to defective hepatic bud formation [41]. Additionally, the lack of HNF1β in mice leads to pancreas agenesis [42]. Maestro et al. suggested that HNF1β is a regulator of pancreas organogenesis and differentiation [43]. HNF1β was also shown to be essential for nephron segmentation during nephrogenesis [44]. Clinically, heterozygous germline mutations in HNF1β cause familial forms of type 2 diabetes called as maturity-onset diabetes of the young, subtype 5 (MODY5), which is frequently associated with congenital abnormalities, such as polycystic kidney, pancreatic hypoplasia and genital tract abnormality [45-47]. Mutations of HNF1β may also be responsible for some sporadic congenital renal abnormalities [48]. In adulthood homeostasis, HNF1β was shown to play a crucial role in renal repair after ischemia/reperfusion in the acute kidney injury model [49, 50].

Contrary to the developmental stage in which HNF1β defect influences normal organ development, overexpression of HNF1β has been reported in multiple malignancies. As stated above, it is specifically expressed in OCCC among ovarian cancers [16-18]. In addition, HNF1β may play an important role in prostate and hepatocellular carcinomas [51-53]. However, the precise mechanism of how HNF1β contributes to cancer biology remains unclear. So far, the reported function of HNF1β includes epithelial morphogenesis and differentiation [54, 55], regulation of the urate transporter and organic anion transporters [56, 57], and the metabolic regulation and ROS reduction discussed here.

## HNF1β DRASTICALLY ALTERS GLYCOLYTIC PROCESS IN OCCC CELLS

The importance of the metabolic aspect of OCCC was predicted as stated above. In addition, there was growing evidence suggesting that HNF1β is associated with glucose metabolism. Balakrishnan et al. and our group reported that HNF1β induces sodium-glucose co-transporter and glucose transporter 1, respectively [21, 58]. Senkel et al. identified 25 target genes of HNF1β in a human embryonic kidney cell line, which includes dipeptidyl peptidase 4 (DPP4), which is known to play a major role in glucose metabolism [59]. Gregori et al. found that the expression of aldolase B, an enzyme that is involved in two opposite metabolic pathways, glycolysis and gluconeogenesis, is controlled by the two HNF1 binding sites in the enhancer [60].

Actually, we noticed a functional link between HNF1β and cell metabolism in our own experiments [21]. We knocked down the expression of HNF1β by shRNA in RMG2 human OCCC cells, which originally has high expression of HNF1β. Then, we found that the growth of HNF1β-high original RMG2 was slower than HNF1β_knockdown RMG2. Nevertheless, we noticed that the color of the media in HNF1β-high original RMG2 tended to be yellow compared to the HNF1β_knockdown RMG2, leading to the finding that lactic acid accumulates more in the former. These results suggest that HNF1β-high RMG2 exhibited higher glucose metabolism, but it was not the consequence of rapid cell proliferation [21]. We further elucidated this seemingly curious phenomenon in detail, and we found that glucose uptake, as well as glycolysis, is significantly higher in HNF1β-high RMG2. These metabolic changes are consistent with “the Warburg effect”. However, the biological influence, perhaps an advantageous influence, of such metabolic alteration for OCCC cells remained unclear. It was not likely that higher glucose uptake directly contributed to cell proliferation, as stated above.

To explore the intracellular metabolic change triggered by HNF1β expression, in the study in this issue of journal, we performed a comprehensive metabolite analysis (the metabolome assay) between HNF1β-high RMG2 and HNF1β_knockdown RMG2 cells [61]. As expected, repression of HNF1β expression caused drastic intracellular metabolic alteration. The most prominent alteration is the increase in glycolysis and, on the other hand, the decrease in oxidative phosphorylation. Because this shift is the main component of the Warburg effect, we can say that HNF1β enhances the Warburg effect in OCCC cells. One question remains: why (for what purpose) do OCCC cells employ such metabolic activity? To answer this question, I will next discuss the biological aspect of the Warburg effect.

## BIOLOGICAL ASPECT OF THE WARBURG EFFECT AND THE ROLE OF HNF1β

Since Warburg first reported unique cancer metabolism, a number of researchers have tried to explain how it contributes to cancer biology. Warburg himself already noticed that this metabolic reprograming does not simply enable cancer cells to live under hypoxic conditions because the Warburg effect is also observed in the environment with abundant oxygen. He speculated that mitochondrial dysfunction in cancer cells hampers oxidative phosphorylation and forced them to switch to glycolysis [62]. However, recent studies have revealed that many cancers do not necessarily have mitochondrial defects [23], or mitochondrial dysfunction does not always linked to Warburg effect [63]. The apparent advantage of energy production by glycolysis is that, if cancer cells have a sufficient glucose supply available, they can produce energy more quickly by glycolysis than by oxidative phosphorylation even under normoxic conditions. Moreover, glycolysis can support cell proliferation in other processes, supplying substances such as nucleotides, amino acids and lipids for biosynthesis. In term of material supply for cell growth, glycolysis is more efficient than oxidative phosphorylation [64-66].

In addition to supporting cell proliferation, the metabolism shift from oxidative phosphorylation to aerobic glycolysis confers another important advantage for cancer cells, namely, cell survival [66, 67]. Suppression of oxidative phosphorylation compromises intrinsic apoptosis through inhibition of proapoptotic proteins, Bax and Bak, and through reduction of ROS production in mitochondria. From this viewpoint, we analyzed the whole metabolic process in HNF1β-high and low RMG2 cells, and obtained several notable findings [61]. First, we could not detect any findings that HNF1β-induced metabolic change contributes to cell proliferation. Second, neither energy nor biomass production seemed augmented in HNF1β-high RMG2. Actually, these were in accordance with the fact that HNF1β_knockdown RMG2 grow more rapidly than HNF1β-high RMG2, as mentioned earlier. Clinically, OCCC is known as a relatively slow-growing tumor, which may explain why it does not receive growth benefit from the Warburg effect. Third, survival of HNF1β-high RMG2 was significantly better compared with HNF1β_knockdown RMG2 especially under hypoxic conditions. Furthermore, these cells were more resistant to CDDP treatment in hypoxic conditions compared with HNF1β_knockdown RMG2. Most importantly, survival benefit of HNF1β-high RMG2 was significantly impaired in low glucose conditions, suggesting that cell survival of HNF1β-high RMG2 is highly dependent of glucose uptake and consumption.

We then measured intracellular ROS activity in these cells, which indicated that HNF1β-high RMG2 had lower levels of intracellular ROS. When these cells were exposed to known extracellular oxidative stressors such as ferric nitrilotriacetate (FeNTA) or H_2_O_2_, HNF1β-high RMG2 had lower ROS activity levels compared with their HNF1β_knockdown counterparts [61].

These data collectively suggest that, HNF1β reduces oxidative phosphorylation, which is the primary source of intracellular ROS, by increasing the oxygen-independent glycolysis, and confers ROS resistance in OCCC cells. Therefore, in this particular case, the Warburg effect primarily contributes cell survival, rather than cell proliferation. Thus, the Warburg effect, which represents frequently observed metabolic alterations in cancers, may comprise of different mechanisms with different biological consequence according to the cancer types and their requirements.

## HNF1β CONFERS ROS RESISTANCE BY MULTIPLE MECHANISMS

In addition to the findings above, our study revealed that HNF1β also contributes ROS reduction with mechanisms other than the Warburg effect [61]. HNF1β-high RMG2 had significantly higher activity of intracellular GSH, a major regulator of intracellular ROS. Furthermore, rBAT, a cystine transporter, was expressed at significantly higher levels in these cells. Knockdown of rBAT by sh-RNA resulted in decreased levels of intracellular GSH and increased ROS activity, suggesting that rBAT plays a major role in HNF1β-triggered ROS resistance. These results strongly suggest that one of the primary roles of HNF1β is to avoid cellular oxidative stress and to enable cells to survive under stressful environments.

There are no reports indicating that HNF1β contributes to ROS resistance. However, Qadri et al. reported that HNF1 and HNF4 mediate hepatic multidrug resistance protein 2 up-regulation during hepatitis C virus gene expression, which may contribute to the cellular detoxification against ROS produced during HCV infection [68]. Wobser et al. reported that suppression of HNF-1 alpha results in mitochondrial dysfunction, cell apoptosis, and increased sensitivity to ceramide [69]. These data suggest that the HNF signal, as a whole, confers ROS resistance and anti-apoptotic effect.

## WHY DOES OCCC SPECIFICALLY UNDERTAKE UNIQUE METABOLIC ACTIVITY AMONG OVARIAN CANCERS?

**Figure 2:**
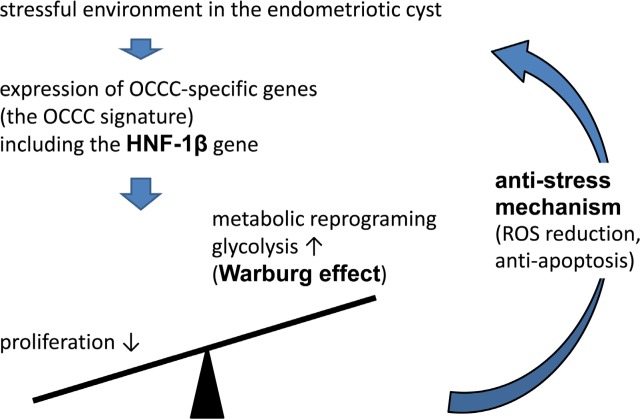
Mechanism that OCCC cope with environmental oxidative stress Oxidative stress induces a set of OCCC-specific genes, which we designated as the OCCC signature. HNF-1b, one of the most important transcriptional factors in the OCCC signature genes, suppresses cell growth on the one hand, and enhances survival mechanism by the metabolic reprogramming on the other hand.

The induction of HNF1β is reasonable in terms of the microenvironment with which these cells have to cope. As shown above, endometrioid epithelial cells are continuously exposed to severe oxidative stress caused by the contents of the cyst [10, 19]. Therefore, the primary requirement for survival of endometrial epithelial cells is the anti-stress capacity rather than proliferative potential. It is likely that HNF1β is induced for this purpose and its expression is maintained throughout malignant progression (Figure 2). HNF1β, as a transcription factor, induces multiple genes which participate in the Warburg effect, but other mechanisms also serve as tools to escape from intracellular stress.

This is particularly important from a clinical point of view because the stress-resistant characteristic of OCCC may lead to the chemo-resistance as we report in this issue. Tamada et al. reported similar findings on CD44, a cell adhesion molecule, which interacted with pyruvate kinase M2 and enhances glycolysis [70]. Ablation of CD44 resulted in the reduction of GSH, the increase in intracellular ROS, and enhanced the effect of chemotherapeutic drugs.

## HNF1β AND SUBSEQUENT METABOLIC ALTERATIONS AS POSSIBLE THERAPEUTIC TARGETS FOR OCCC

Currently, most of the cancer chemotherapies target cell proliferation. However, with growing evidence accumulating that indicates the importance of cancer-specific metabolism, attempts to use modification of cancer metabolism as therapeutic target are also being focused [71, 72]. PI3K pathway, one of the most commonly activated signaling pathways in cancers including OCCC, has recently been implicated in glycolytic activity [73-75]. Activated Akt signal was shown to stimulates aerobic glycolysis and maintain cell survival and resistance to apoptosis [31, 76]. It is assumed that oncogenes/tumor suppressor genes are more or less linked to the metabolic activity of cancer cells [30-34, 70, 71, 77]. Therefore, molecular target therapy against these signals might modulate intracellular metabolism toward anticancer effect [30, 78, 79].

More specific strategies to target metabolism itself are also being developed. PKM2, an isoform of PK, which catalyzes the conversion of phosphoenolpyruvate to pyruvate, is known to play a key role in glucose metabolism, and several small-molecule PKM2 activators are being developed. Likewise, inhibitors of phosphoglycerate mutase 1 (PGAM1) and mutant isocitrate dehydrogenase (IDH), both catalases involved in glycolysis in cancer cells, are considered to be promising candidates as cancer drugs [80].

Because it has been reported that the Warburg effect as well as other metabolic alterations affect mitochondrial function, ROS production, and eventual apoptosis, each step involved in the oxidative phosphorylation is also a candidate target. It has been shown that suppression of glycolysis and shift to mitochondrial oxidation leads to cancer cell death [81].

**Figure 3:**
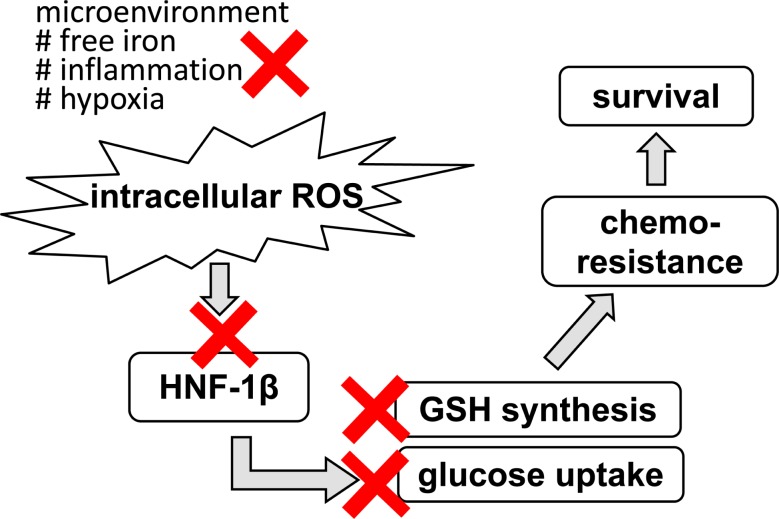
Potential therapeutic target for OCCC in terms of stress response Primarily, improvement of stressful environment by the surgical intervention may lead to inhibition of cancer occurrence. In addition, interventions into each step of HNF-1β-related survival mechanism may provide more specific targeted therapy.

Our study on the metabolic alterations caused by HNF1β in OCCC evokes some hints to disease management, as each step of clarified alterations could be candidate targets for treatment (Figure 3). First, we demonstrated that oxidative stress in the endometriotic cyst has causative relationship to OCCC [19]. Improvement of such microenvironment by surgical treatment may reduce the risk of cancer development. Second, we showed that HNF1β augments cell survival by enhancing ROS resistance in OCCC. HNF1β inhibition with some type of inhibitor, such as the microRNA mir-802 [82], may have a therapeutic effect by abrogating ROS resistance. Third, our data indicated that HNF1β-induced cell survival is dependent on the glucose supply. HNF1β-high RMG2 cells were more vulnerable to glucose deprivation. Several reports suggested that glucose deprivation is critical in cancers displaying the Warburg effect [72, 83, 84]. Therefore, glucose metabolism may be a therapeutic target in OCCC with high HNF1β expression. In ovarian cancer, metformin, an anti-diabetic drug, was shown to inhibit cancer growth and increase paclitaxel sensitivity in mouse model [85]. More specific reagents were also indicated to have anti-tumor effect by altering glycolytic activity [86-88] *in vitro* and in animal experiments. Tamada et al. showed that modulation of glucose metabolism by CD44 contributes to the antioxidant status and drug resistance in cancer cells [70]. Human trial, especially from large studies, are not available yet, and to be evaluated in future. Finally, inhibition of GSH synthesis using reagents such as buthionine sulfoximine [89, 90], or inhibition of GSH synthesis by targeting the cystine transporter rBAT, may enhance the effect of conventional chemotherapy.

## CONCLUSIONS

Our studies along with others have started to explore the importance of cancer-specific metabolism in pursuing the cancer biology and also therapeutic application. Our serial studies suggest the possibility that cells maneuver their own metabolism to survive under stressful environments, and cancers that arise in such stressful conditions exhibit metabolic activity fine-tuned to resist intracellular stress, which may, in turn, leads to the drug resistance.

Cancer specific metabolic activity could be a promising target as an individualized treatment in various cancers, and for this purpose, clarifying the features of metabolic activity in each cancer is needed.

## Abbreviations

HNF1β: hepatocyte nuclear factor 1β
OCCC: ovarian clear cell carcinoma
ROS: reactive oxygen species
